# The Impact of Mobility Restriction Strategies in the Control of the COVID-19 Pandemic: Modelling the Relation between COVID-19 Health and Community Mobility Data

**DOI:** 10.3390/ijerph181910560

**Published:** 2021-10-08

**Authors:** Adil Al Wahaibi, Amal Al Maani, Fatma Alyaquobi, Abdullah Al Manji, Khalid Al Harthy, Bader Al Rawahi, Abdullah Alqayoudhi, Sulien Al Khalili, Amina Al-Jardani, Seif Al-Abri

**Affiliations:** Directorate General for Disease Surveillance and Control, Ministry of Health, P.O. Box 393, Muscat 113, Oman; amalsaifalmaani@gmail.com (A.A.M.); fatmayaquobi@gmail.com (F.A.); doctoraway11@gmail.com (A.A.M.); khalidsaidharthy@gmail.com (K.A.H.); baderalrawahi4@gmail.com (B.A.R.); alqaudi99@gmail.com (A.A.); sulienkhalili18@gmail.com (S.A.K.); aksaljardani@gmail.com (A.A.-J.); salabri@gmail.com (S.A.-A.)

**Keywords:** Oman, COVID-19, SARS-CoV-2, mobility restrictions, non-pharmaceutical interventions, pandemics, non-linear distributed lag model

## Abstract

Background: Non-pharmaceutical interventions (NPIs), particularly mobility restrictions, are mainstay measures for the COVID-19 pandemic worldwide. We evaluated the effects of Oman’s mobility restriction strategies to highlight their efficacy in controlling the pandemic. Methods: Accessible national data of daily admissions and deaths were collected from 1 April 2020 to 22 May 2021. Google Community Mobility Report (CMR) data were downloaded for the same period. Among six CMR categories, three were used and reduced to one index—the community mobility index (CMI). We used a generalised linear model with a negative binomial distribution combined with a non-linear distributed lag model to investigate the short-term effects of CMI on the number of admitted PCR-confirmed COVID-19 cases and deaths, controlling for public holidays, day of the week, and Eid/Ramadan days. Results: We demonstrated the feasibility of using CMRs in the evaluation and monitoring of different NPIs, particularly those related to movement restriction. The best movement restriction strategy was a curfew from 7 p.m. to 5 a.m. (level 3 of CMI = 8), which had a total reduction of 35% (95% confidence interval (CI); 25–44%) in new COVID-19 admissions in the following two weeks, and a fatality reduction in the following four weeks by 52% (95% CI; 11–75%). Conclusion: Evening lockdown significantly affected the course of the pandemic in Oman which lines up with similar studies throughout the world.

## 1. Introduction

The transmission of SARS-CoV-2, the virus which causes COVID-19, occurs mainly through contact (direct/indirect) and droplets. Its transmission is influenced by many factors including human mobility patterns and contact rates [1]. Many of the affected countries have implemented non-pharmaceutical interventions (NPIs) to help in the battle of this prolonged war against the disease. NPIs adopted from country to country have ranged from airport closures, restriction of movements through different movement restriction strategies to enforcement of social distancing and mandatory mask wearing in public places [2,3,4,5]. 

The population mobility restriction interventions at different phases of the pandemic were one of the main NPIs exercised by governments with the main objective of flattening the curve and slowing the speed of transmission. These mobility interventions have been especially crucial in the pre-vaccination era. Many studies and data analysing the role of NPIs implemented in many countries as well as epidemiological models have highlighted that public and social gathering restrictions, remote working and schooling, and curfews are the most effective public health measures in control the course of the pandemic [6,7,8].

As a response to the COVID-19 pandemic, the Omani government has established a supreme committee represented by ministers from different sectors, including the ministry of health, representing technical expertise. This committee reviewed the epidemiological reports on the pandemic for the country and makes decisions for pandemic control in the form of many NPIs, including alterations between the closure and opening of points of entries, schools, mosques, commercial activities, and the introduction of movement restriction based on the pandemic situation and saturation of the Omani health care system [3]. 

Mobility restriction strategies in Oman were implemented at various times (curfew from 7 p.m., 8 p.m., and 9 p.m.) and durations (curfew for one week or two weeks). In this study, we investigated how mobility restriction decisions in Oman have influenced the community’s behaviour, reflected through mobility changes, to limit person-to-person interactions. The impact of the mobility changes on the disease burden was analysed using COVID-19 admission and mortality data to inform the best future restriction strategies in Oman for controlling the pandemic. 

## 2. Methodology

To study the effects of mobility restrictions, we used two sets of data: Google Community Mobility data (CMR) and Oman’s official COVID-19 data (admissions and deaths). The major NPIs concerning mobility restrictions in Oman were identified and reflected in the CMR data. Then, the CMR data were categorised into levels reflecting the different grades of the mobility restriction measures. These levels were modelled against the admissions and deaths, taking into consideration the lag effects of the mobility restriction measures on the admissions or deaths from COVID-19. 

### 2.1. Health Data

Freely accessible national data regarding COVID-19 in Oman were used for the period of 1 April 2020 to 22 May 2021. The data included pertained to PCR-confirmed COVID-19 daily admissions and deaths. 

### 2.2. CMR

Daily Google Community data were downloaded for the same time period as was the surveillance data [9]. These data are collected by Google via users’ smartphones or handheld devices that allowed and enabled location services indicating their activities [10]. Percentage changes of these activities from the baseline (pre-COVID-19 periods) are provided for the following categories: ‘retail and recreation’, ‘parks’, ‘workplaces’, ‘residential’, ‘grocery and pharmacies’ and ‘transportation use’. These percentage changes represent the relative changes compared to the baseline, not the actual number of users. ‘Parks’ activities were not included in our analysis because this indicator has strong anticipated seasonal changes [8]. ‘Transportation’, which mainly indicates the use of public transport and train hubs, was not included in our analysis because such modalities of transport are not well-developed in Oman. Finally, ‘residential’ activity was not used as it is not indicative of activity [8]. Therefore, we studied the following CMR activities: ‘retail and recreation’, ‘workplaces’, and ‘grocery and pharmacies’. 

### 2.3. Descriptive Statistics and Correlation

Health and CMR data were described by mean, median, maximum, and minimum values. The Pearson correlation between the studied CMR categories and health data was completed.

### 2.4. CMR and Oman’s Mobility Restriction NPIs

NPIs that were decided upon by the Omani Supreme Committee for COVID-19, especially those concerning movement restriction, were retrieved from the Omani media [11] and plotted against the CMR data. Because they are highly correlated, the three CMR indices were reduced to one index: the community mobility index (CMI), using principal component analysis (PCA). The first component of PCA, which captures the maximum variance of the studied CMR indices, was selected. The time series of this CMI was plotted against the selected mobility restriction NPIs. This enabled the categorization of the CMI levels to reflect the levels of movement restriction. 

### 2.5. Modelling 

In this study, we modelled the daily admissions/deaths (as a dependent variable) with the CMI (as an independent variable) controlling for the time trend (smoothed using natural cubic spline of numerical date with seven degrees of freedom) and public holidays, day of the week, and religious events (Ramadan and Eid) as factors.

Because daily admissions and daily deaths are count data, we used a generalised linear model (GLM) with a Poisson distribution. However, to control for the data overdispersion, we changed the distribution of the GLM to the negative binomial distribution. 

To account for the delayed effects between the daily mobility data (CMI) and the daily admissions/deaths, we incorporated the CMI into a non-linear distributed lag model (NLDM) allowing the effects of CMI to be distributed over a lag period in the model. This was conducted through the ‘cross-basis’ function in the (DLNM) package in R 4.0.2 software [12], which models both the lag period and exposure (effects of CMI on admissions/deaths) in one component. In the ‘cross-basis’ function, we used a natural cubic spline with four degrees of freedom for the lag component and a polynomial spline for the exposure component. Such an approach for the choice of cross-basis parameters was also adopted by Sulyok M et al. [8]. For the lag period, we examined a lag of 14 days for the daily number of admissions and 28 days for deaths. These were chosen to explore the effects on more extended periods [8]. The reference value in the NLDM was the median CMI value representing times of relaxed control measures. The resulting NLDM was then used in the GLM instead of CMI. All analysis was performed using ‘DLNM’ [12] and ‘MGCV’ [13] packages in R 4.0.2 software [14].

### 2.6. Model Validation and Sensitivity Analysis

Sensitivity analysis was carried out to explore model assumptions and robustness through changing the constraints of the exposure component of the DLNM from ‘polynomial’ to ‘natural cubic spline’ and changing the degree of freedom of the lag component of DLNM natural cubic spline to three and five. All these parameters were changed, and the best model was chosen based on the lowest Akaike information criterion (AIC).

In addition, many time-related co-factors were tried in the models, such as public holidays, day of the week, and Ramadan/Eid times. The selection of the best model was based on AIC through the function ‘stepAIC’ in R which favours holiday days in the admission model and Ramadan/Eid days for the mortality model. 

As the study used freely accessed health data and open source CMR, there was no need for ethical approval. 

## 3. Results

**Figure 1:**
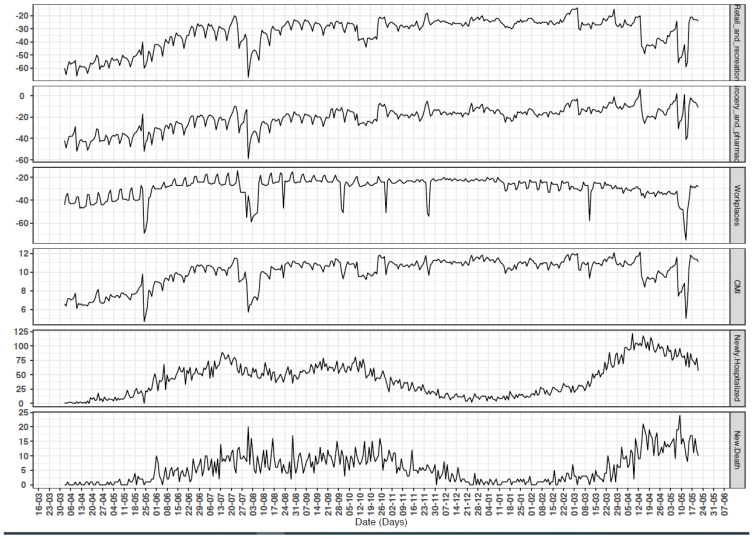
Time series of the studied community mobility reports (CMRs), community mobility index (CMI), hospitalisation and deaths from COVID-19, Oman, 1 April 2020 to 22 May 2021.

Time series data of ‘retail and recreation’, ‘grocery and pharmacy’, and ‘workplaces’ show similarity in the trends of percentage changes compared with CMI as shown in Figure 1. This similarity was also supported by the strong correlation between these indices using Pearson correlation analysis (see Supplementary Material Figure S1). Figure 1 also illustrates the time series of the number of COVID-19 new admissions and deaths from 1 April 2020 to 22 May 2021. Two waves of the pandemic are evident: the first wave started at the beginning of May 2020 and ended in late November 2020. The second wave started at the beginning of March 2021, and it is still active, with some decline by the end of April 2021. 

The median CMI, used as a reference point (baseline) in this study, was 10.5. The minimum and maximum CMI were 4.7 and 12.2, respectively. The maximum number of admissions was 122, and the maximum number of deaths was 24. The minimum for both was zero admissions and fatalities, Table 1. 

**Figure 2:**
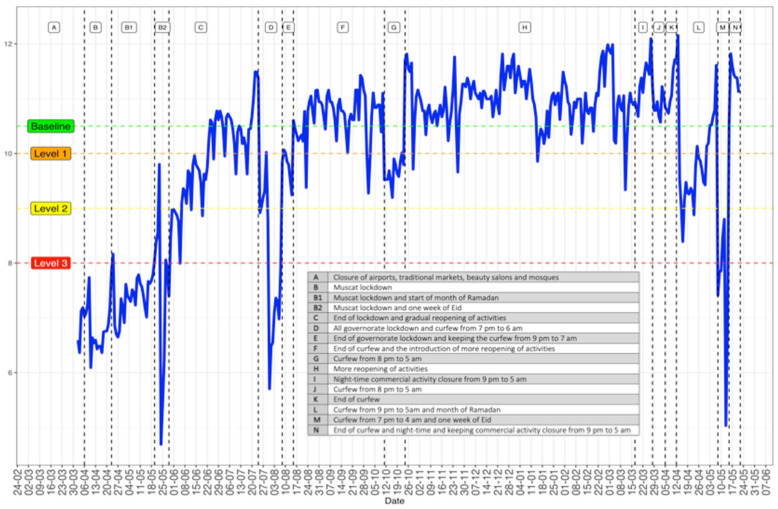
Omani government-mandated movement restriction NPIs, plotted against the CMI levels with an illustrative table of each mobility control measure. The weekly sharp troughs in the CMI line represent weekends and public holidays.

The NPIs, mainly movement restriction measures, were plotted against the CMI levels, as shown in Figure 2. Assigning the baseline at 10.5, we labelled the remaining levels of CMI to capture the different strategies of movement restriction. Apart from weekends and public holidays, level 1 (CMI at 10) was reached in (E), which was a one-week curfew from 9 p.m. to 7 a.m. and (G), which was a two-week curfew from 8 p.m. to 5 a.m. Level 3 (CMI at 8) was reached with the Muscat (the capital of Oman) lockdown during the month of Ramadan and the Eid holiday in May 2020, (B). In addition, level 3 was reached during lockdown in all governorates with a two-week curfew from 7 p.m. to 6 a.m. (D) and during the one-week curfew from 7 p.m. to 4 a.m. during Eid (M) in May 2021. To investigate the gradient effects of the CMI on COVID-19 numbers of admissions and mortality, we introduced level 2 at a CMI of 9. This level was reached during the 9 p.m. to 5 a.m. curfew for the entire month of Ramadan 2021 (L). 

Figure 2 also indicates that for an isolated movement restriction (that is not a descaled restriction from longer to fewer hours) and an exact timing, the number of restriction days creates different effects on the CMI. For example, in the two-week curfew from 8 p.m. to 5 a.m. in (G), the CMI has reached below level 1, whereas the one-week curfew for the exact timings has not affected the CMI even below the baseline (J). Note also that the night closure of commercial activities (I) did not decrease the CMI but increased it more than the baseline. 

**Figure 3:**
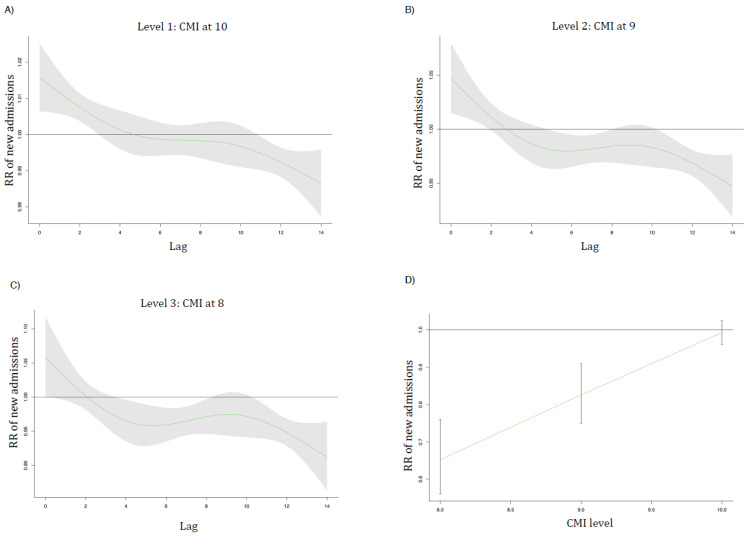
The lag curves (in days) representing the relative risk (RR) of new admissions with a shaded 95% CI for each future day following a decrease in CMI at level 1 (**A**), level 2 (**B**), and level 3 (**C**). (**D**) represents the overall reduction in hospitalization with each level of CMI. Level 1 is CMI = 10, level 2 is CMI = 9, and level 3 is CMI = 8.

**Figure 4:**
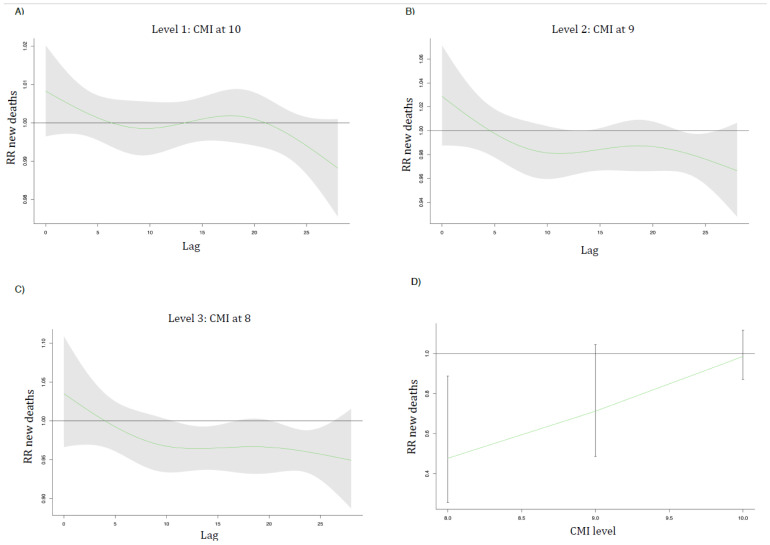
The lag curves (in days) representing the RR of COVID-19 mortality with a shaded 95% CI for each future day following a decrease in CMI at level 1 (**A**), level 2 (**B**), and level 3 (**C**). (**D**) represents the overall reduction in deaths with each level of CMI. Level 1 is CMI = 10, level 2 is CMI = 9, and level 3 is CMI = 8.

Figure 3 and Figure 4 show the lag curves (in days) representing the association between admissions/deaths relative risk (RR) with a shaded 95% CI for each future day following a decrease in CMI at level 1 (A), level 2 (B), and level 3 (C). In (D), the overall reduction in admissions/deaths for each level of CMI is presented. 

For admissions, Figure 3 shows that the statistically significant reduction in RR occurred slightly on day six but was more evident on day 14 from the start of the CMI reduction with RR = 0.95 (95% CI; 0.92–0.98) for level 2 and RR = 0.91 (95% CI; 0.86–0.96) for level 3. Overall, reducing CMI to level 2 (CMI = 9) causes a 17% reduction in admissions, RR = 0.83 (95% CI: 0.70–0.91), whereas reducing it to level 3 (CMI = 8) causes a 35% reduction in admissions, RR = 0.65 (95% CI: 0.56–0.75), Figure 3D. 

On the other hand, the effect of CMI reduction on mortality of COVID-19 is subtle. The maximum effects occurred from day 20 to 25 for the reduction in CMI. For example, for level 3 (CMI = 8), CMI reduction in the RR of death on day 24 was estimated to be 0.96 [95% CI: 0.93–0.98]. The overall effects of CMI on the reduction in mortality from COVID-19 are only statistically significant at level 3 (CMI = 8) with RR = 0.48 (0.25–0.89).

## 4. Discussion 

Our study showed the feasibility of using the CMR in the evaluation and monitoring of different NPIs, particularly those related to movement restriction. We found that the best movement restriction strategy was a curfew from 7 p.m. to 5 a.m. (level 3 of CMI = 8), which coincided with a 35% (95% CI; 25–44%) reduction in new COVID-19 admissions in the following two weeks and a much wider scale reduction in deaths in the following four weeks (52% (95% CI; 11–75%)). 

Similar to our findings, the positive impact of curfew in admissions was demonstrated in a mathematical modelling study carried out in France where it was estimated that curfew measures allowed hospitalizations to plateau [15]. In addition, the implementation of lockdown in Hubei Province in January 2020 showed a reduction in the confirmed cases and daily increments in incidence in 16 regional-level municipalities after 9 to 12 days [16]. Supporting our results on the effects of mobility reduction on mortality, Hadjidemetriou et al. showed that mobility reduction significantly reduced COVID-19-related deaths in the United Kingdom [17]. On the other hand, a study conducted in Germany and Switzerland did not find evidence that curfews are more effective than banning groups of more than two individuals for reducing fatality rates [18]. 

Curfews, lockdowns, and closing/restricting places where people gather in small or large numbers for extended periods are considered highly effective in controlling the pandemic. However, the effectiveness of these measures varies between countries and depends on the local context. This includes the stage of the pandemic as well as the socio-economic, cultural and, political characteristics of the country [19]. For example, in our study, some interventions such as commercial activity closure from 8 p.m. had, in fact, increased the mobility above our baseline as individuals were rushing to finish their shopping prior to closure time, especially for the imminent religious holidays. It is worth noting that the overall compliance of the Omani population with the national restriction measures, shown by the degree of temporal coherence in mobility changes with the different NPIs (Figure 2), is supportive of a unified national governmental approach in leading the NPI against the pandemic.

In our study, we found that the effect of mobility restriction on admission is level-dependent. That is, the more the reduction in the CMI, the more the reduction in admissions and deaths. Similar to our findings, this pattern was seen in the United Kingdom, where the introduction of tier 3 (very high alert) restrictions were associated with a further 14% (95% CI 10% to 19%) reduction in infections compared to tier 2 (high alert) restrictions [20].

Our study found that lockdown has prevented 35% (25–44%) admissions in two weeks. These results are comparable to a model analysis conducted for one month, which has found that lockdown prevented 87% of admissions [21]. A cross-country study in France estimated that the maximum effects of the lockdown policies in reducing COVID-19 admissions were evident within ten days; this is similar to our findings, which indicated the impact happening from 6 to 14 days [22].

In this study, the effects of mobility restriction on mortality were not as pronounced and significant as those of admissions represented by wider CIs. COVID-19 mortality, as a constant reliable metric for pandemic response to movement restrictions, might have its own limitations. It is mainly dependent on the treating hospital, treatment regimen, severity of the cases, and even initial classification of the death as COVID-19-related. In addition, such measures as curfews may have unexpected and negative outcomes, such as limiting access to health care facilities with major impacts on patient health and chance of survival in some cases as reported by Haug et al. [19]. 

Similar studies have evaluated the use of proxy data to measure either the adoption of NPIs by people or the mobility changes of people due to these NPIs. The former was carried out, for example, by using Facebook data to measure social connectedness as a surrogate of social distancing [23] or using Wikipedia and Reddit responses to NPIs [24]. The latter, which uses mobility changes as a proxy to measure NPIs, was carried out through using Google CMRs [25,26], Teralytics mobility data [27], University of Maryland COVID-19 impact analysis platform [28], and Facebook [29].

As a limitation of this study, the use of CMRs might not be a representative index in measuring the effects of all NPIs that were adopted by the Omani government. However, we tried to overcome this by concentrating only on the evaluation of mobility restriction. Another limitation of the study is that the CMR data only existed from February 2020, and there was a need to compare the CMR data in religious events (such as Eid and Ramadan) with the pre-COVID-19 years. Although we tried to overcome this limitation by controlling for these events in the main GLM model, future studies are needed to effectively control for the effects of these events on the course of a pandemic.

The effects of vaccination were not controlled for, which might also be a limitation of this study. However, although vaccination started in early January 2021, it covered only 4% of the population by the end of May due to the scarcity of vaccines. The larger-scale vaccine rollout, which started in early June 2021, aims to target 70% of the population by the end of 2021 [30]. 

This is the first study conducted in the Middle East to quantify and evaluate the effects of mobility restriction measures and to determine the best NPI approach in controlling the increasing numbers of admissions and deaths. It will help decision-makers in refining the management of COVID-19 on a larger scale, protecting the public from COVID-19, and preserving the economy and social integrity of the population. Nevertheless, lockdown itself has its own negative impacts on the psychological [31] and financial well-being of individuals and countries [32]. Therefore, a comprehensive public health approach to pandemic management should be taken with these lockdowns, including the provision of financial and logistics support, providing home care and monitoring for sick individuals, and self-reporting through artificial intelligence with a call for help linked to community health teams and ambulance systems. In the absence of this comprehensive public health response system, mobility restriction interventions may drive more adverse outcomes than intended benefits. 

## 5. Conclusions

Our study suggests that lockdown, as a form of NPI, effectively reduces hospitalization and deaths due to COVID-19. However, to sustain such intervention, we need to address its other adverse social, economic, and health effects. Such aspects of the impact of these curfews need to be investigated in future prospective studies. 

## Figures and Tables

**Table 1:** Descriptive statistics of the CMR, CMI, hospitalization and death.

	Mean	Median	Min	Max
Retail and Recreation	−33.2	−28.0	−67.0	−14.0
Grocery and Pharmacy	−21.1	−18.0	−59.0	6.0
Workplaces	−29.0	−26.0	−75.0	−14.0
CMI	10.0	10.5	4.7	12.2
Newly Hospitalised	42.3	42.0	0.0	122.0
New Death	5.7	5.0	0.0	24.0

CMI: community mobility index.

## Data Availability

Publicly available datasets were analyzed in this study. This data can be found here: https://play.google.com/store/apps/details?id=om.gov.moh.tarassudapplication&hl=en&gl=US (accessed on 5 October 2021) and https://www.google.com/covid19/mobility/ (accessed on 5 October 2021).

## Supplementary Materials

The following are available online at https://www.mdpi.com/article/10.3390/ijerph181910560/s1, Figure S1: Pearson correlation between studied CMR, CMI, admission number and deaths. The red colour represents negative correlations and the blue colour represents positive correlations. The intensity of the colour represents the strength of the correlation. The numbers inside the boxes are the *p*-values for the correlation.
