# Supplementary material for: The Impact of Mobility Restriction Strategies in the Control of the COVID-19 Pandemic: Modelling the Relation between COVID-19 Health and Community Mobility Data
Source: Int J Environ Res Public Health. 2021 Oct 8;18(19):10560. doi: 10.3390/ijerph181910560 (PMC8508456; doi:10.3390/ijerph181910560)

Supplementary Figure S1. Pearson correlation between studied CMR, CMI, admission number and deaths. The red colour is negative correlation and blue colour is positive correlation. The intensity of the colour represents the strength of the correlation. The numbers inside the boxes are the P-values for the correlation.

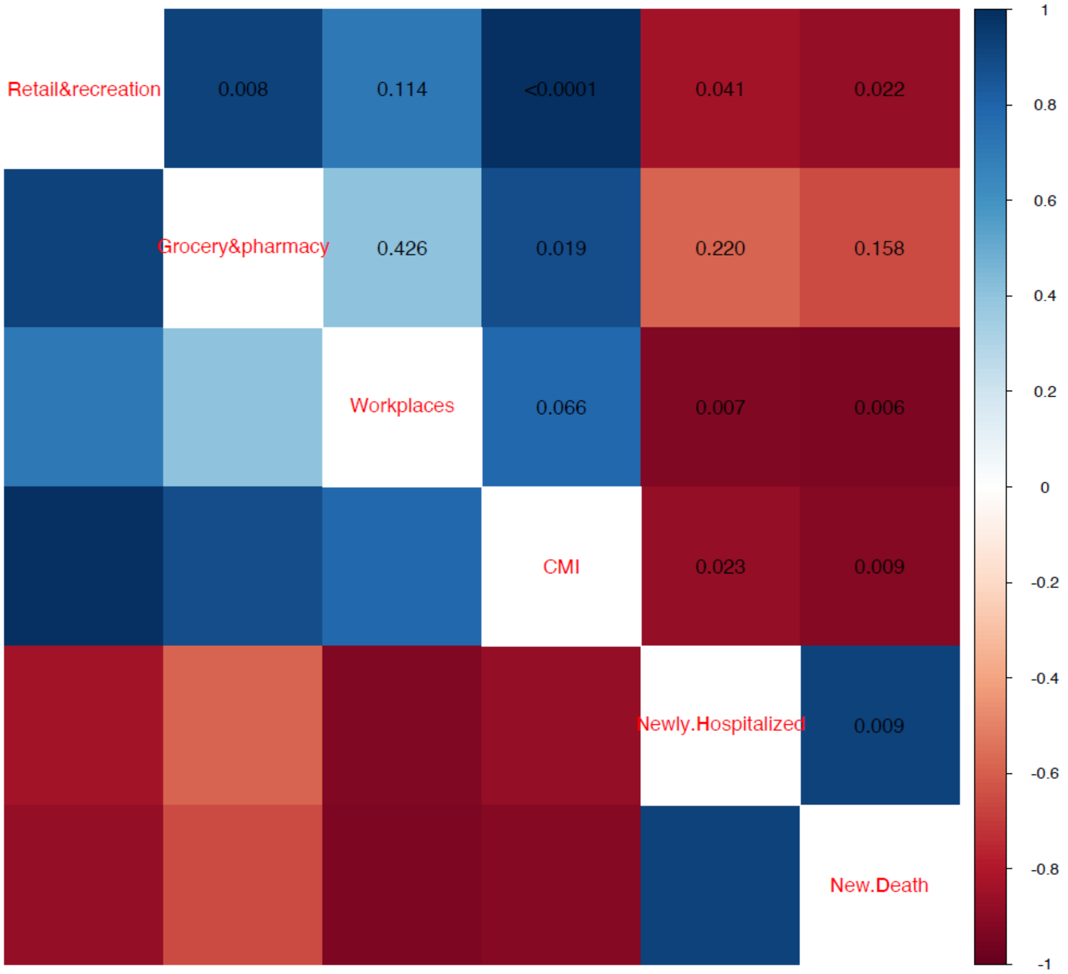

Supplement: Supplementary file 1 [file ijerph-18-10560-s001.zip › ijerph-1412335-supplementary.pdf]
